# Accreditation in continuing veterinary education: development of an accreditation system and selection of accreditation criteria

**DOI:** 10.3389/fvets.2023.1181961

**Published:** 2023-07-27

**Authors:** Maria Kareskoski

**Affiliations:** ^1^Department of Production Animal Medicine, Faculty of Veterinary Medicine, University of Helsinki, Helsinki, Finland; ^2^Veterinary Continuing Education in Europe (VetCEE), Brussels, Belgium

**Keywords:** continuing education, veterinary medicine, accreditation, continuing professional development, accreditation criteria

## Abstract

With the increased supply and demand of veterinary continuing education (CE) and the growing number of CE providers, a clear need has arisen for a multinational accreditation system for veterinary CE. The objective of this document is to describe the current state of veterinary CE accreditation and the development of an accreditation system for veterinary CE, and discuss accreditation criteria and their pedagogical and practical significance. The hypothesis is that a profile of essential, pedagogically grounded, accreditation criteria can be established and utilized effectively in quality assessment. Accreditation criteria for veterinary CE can be created based on four selection principles: educational minimum requirements, coherence, efficacy, and assessability. The selected educational quality criteria are related to needs assessment, correlation of target audience and level of instruction, definition of scope, workload, and number of credits, organizer and instructor qualifications, constructive alignment, assessment of learning, learner engagement, and scientific quality of CE content. The created accreditation criteria and protocols should be regularly re-evaluated and modified in close collaboration with the relevant stakeholders. The desired outcome of CE, including behavior change and improvement of practice and ultimately human and animal health, remains challenging to predict based on course descriptions by the providers, and further research is needed.

## 1. Introduction

The vast accumulation of knowledge, or knowledge explosion, in veterinary medicine has led to an increased need for limiting the content of teaching in basic veterinary education to the bare essentials considered Day One Competencies (1–3). The aim of restricting the content is to ensure adequate learning of essential knowledge and skills within the workload and time restraints (4). When core content is chosen for veterinary education curricula, some areas are left out or addressed only minimally. This leads, in turn, to an increased need for postgraduate training and professional development (CPD) in veterinary medicine, as graduated veterinarians require further education to supplement the basic veterinary skills acquired during their veterinary training (2).

Continuing education (CE) is considered one element of CPD, and it can take many forms beyond that of formal course work. In some countries, regular CPD is a requirement for maintenance of certification for graduated veterinarians by their national licensing bodies. In the United Kingdom, all practicing veterinary surgeons in the Royal College of Veterinary Surgeons (RCVS) Register must complete a minimum CPD requirement currently set at 35 h a year by The RCVS Code of Professional Conduct for Veterinary Surgeons (5). Besides meeting requirements, CE is associated with clear benefits, such as improved confidence and motivation, and opportunities for transferable credits accumulating into individually tailored, flexible mid-tier qualification pathways. Recent graduates have been reported to frequently lack confidence in their own skills and abilities (6, 7), and this mindset coupled with a large workload can prove quite distressing and tiring for the new practitioner (1), indicating a need for further training and support.

The increased demand of veterinary CE and the advancements in digital technology had already begun to attract new course providers into the field of veterinary education in the 2000’s (8). Coupled with the sudden and widespread utilization of digital learning at the start of the COVID-19 pandemic and lockdowns, the supply of webinars and distance learning started to increase dramatically (9). With this potentially lucrative niche of offering CE webinars and courses, growing concerns appeared regarding the educational quality of CE, and the qualifications of providers in organizing useful, evidence-based CE without undue commercial influence by the pharmaceutical industry or other operators in veterinary medicine.

With the increased supply and demand of veterinary CE and the growing number of CE providers, a clear need has arisen for a multinational accreditation system for veterinary CE in Europe. The objective of this text is to first briefly consider the current state of veterinary CE accreditation in Europe, and then describe the development of an accreditation system. The pedagogical background for selected accreditation criteria is discussed. The working hypothesis is that a profile of essential, pedagogically grounded and practical accreditation criteria can be established and utilized effectively as an assessment matrix or checklist for quality assessment, but continuous re-evaluation and quality assurance are needed.

## 2. Definition and purpose of accreditation

Accreditation of CE entails the assessment of the educational quality of a program, in order to ensure that the programs meet appropriate standards of quality. Leist (10) described accreditation as “a *method for maintaining integrity of the educational structure, process, and outcomes*.” Accreditation raises the standard for CE and improves the quality of health care, and accreditation is a way of ensuring that the providers are credible and competent (10). In medical CE, improving patient care is the overarching purpose of CE accreditation (11).

In the information overload of today’s world, with the abundance of providers of varying quality, the significance of accreditation of veterinary CE is definitely on the rise. For veterinary graduates planning their personal CPD path, accreditation means quality assurance, and that certain quality standards have been assessed and are met. It can also be a way of ensuring that any credits attained can be accepted as CPD for maintenance of certification, or as part of flexible pathways of CPD with possibly achieving eligibility to take an international certification examination in a veterinary specialty, instead of completing a structured residency program at a certified institution.

The role of accreditation in relation to the providers is one of guidance and documentation, but also of rewarding and supporting providers in developing their CE and increasing the visibility of accredited, reliable providers and courses. Through setting standards for educational activities, the accreditation body can provide a framework by which providers plan, implement, and evaluate CE (11) and thus establish common guidelines for improving educational quality and helping veterinarians improve their practice.

## 3. Current state of veterinary CE accreditation

Accreditation of CE in veterinary medicine has developed alongside similar advancements in the medical CE field. Accreditation of medical CE providers began in the late 1960s when new knowledge and innovations created a need for CPD, and quality assurance was becoming an important concept in health care (10). The American Medical Association (AMA) established criteria for medical CE accreditation in 1968, and the current accreditation body, the Accreditation Council for Continuing Medical Education (ACCME) was formed in 1981 (10). The ACCME accredits organizations that provide medical CE, not individual educational activities (12). In 2016, the ACCME published a set of criteria for Accreditation with Commendation to further encourage providers to develop their educational quality in terms of pedagogy, engagement, evaluation, and change management (12).

In the veterinary profession, the Association of American Boards of Examiners in Veterinary Medicine (later formed into the current American Association of Veterinary State Boards, AAVSB), was formed in 1957 (13). The AAVSB formed the Registry of Approved Continuing Education (RACE) in 1997 (14), with the purpose of developing and applying uniform standards for CE providers and programs in veterinary medicine (14). The AAVSB states that the RACE program reviews and approves programs but does not provide accreditation for CE providers (14), while ACCME specifically accredits organizations and not individual activities (12). The RACE approval and credit system has become well established and widely recognized, perhaps even a gold standard, of veterinary CE accreditation, but is of somewhat limited use for veterinarians outside of the US. The AAVSB also offers veterinary professionals in the US a free online service (RACEtrack) for recording CE credits (14).

In Europe, the CE field has been based on the use of transferable credits and recognition of qualifications via the European Credit Transfer and Accumulation System (ECTS), and has relied mostly on national accreditation or licensing bodies. In the United Kingdom, the Royal College of Veterinary Surgeons (RCVs) monitors CPD programs of veterinarians, and provides a digital CE recording platform, 1CPD, for United Kingdom practitioners (5). The Akademie für tieraerztliche Fortbildung (Academy for Veterinary Continuing Education, ATF) of the Bundestierärztekammer (Federal Chamber of Veterinary Surgeons, BTK) is responsible for the certification of CE courses for veterinarians in Germany. The ATF accreditation corresponds to the RACE accreditation in the US and the CE rules of the RCVS in the United Kingdom, and the Veterinary Council of Ireland. Most countries also offer their own national veterinary specialization programs producing a variable, country-specific framework of CE recognition. In Australia and New Zealand, the Australasian Veterinary Boards Council serves a similar purpose as the AAVSB or the RCVS (15).

## 4. Development of a European veterinary CE accreditation system

Participation in CE for maintenance of certification is required in many countries, but not always effectively monitored or recorded. In European countries, CE credits can be recorded by the licensing bodies, but these mainly record credits without reviewing the content or quality of CE. There has also been a lack of consensus on the grounds of accreditation, as various organizations or national authorities have created their own accreditation criteria, or simply register credits according to the number of hours dedicated to CE. As flexible pathways to veterinary specialization become available, accredited, high-quality CE becomes an essential part of individual CPD planning and implementation.

To fulfill the need for common, established accreditation criteria and CE services for both practitioners and providers, the Veterinary Continuing Education in Europe (VetCEE) was founded in 2014. VetCEE is an international non-profit association, and was formed as a joint initiative of veterinary academia (European Association of Establishments of Veterinary Education, EAEVE), veterinary specialists (European Board of Veterinary Specialization, EBVS) and the veterinary profession (Federation of Veterinarians of Europe, FVE, and Union of European Veterinary Practitioners, UEVP). The main purpose of VetCEE is to provide accreditation of national and international programs for veterinary CE in Europe and to facilitate the recognition of CPD between the various countries. Awarding titles and recording CE credits remains under the responsibility of the national authorities or statutory bodies (16), and VetCEE specifically evaluates and accredits CE courses or other educational activities, not providers or organizations.

Since 2014, VetCEE has developed its protocols and internal rules, and accredited a limited number of veterinary CE. After significant changes to its application systems, evaluation protocols, and accreditation criteria, VetCEE launched its improved accreditation system in 2022, with the aim of providing better services for veterinary practitioners and providers. Quality evaluation and content peer review by external evaluators with specific competencies in different fields of veterinary medicine form the basis for CE evaluation in the VetCEE system.

## 5. Selection of accreditation criteria in veterinary CE

### 5.1. Justifications for selection of criteria

**Figure 1:**
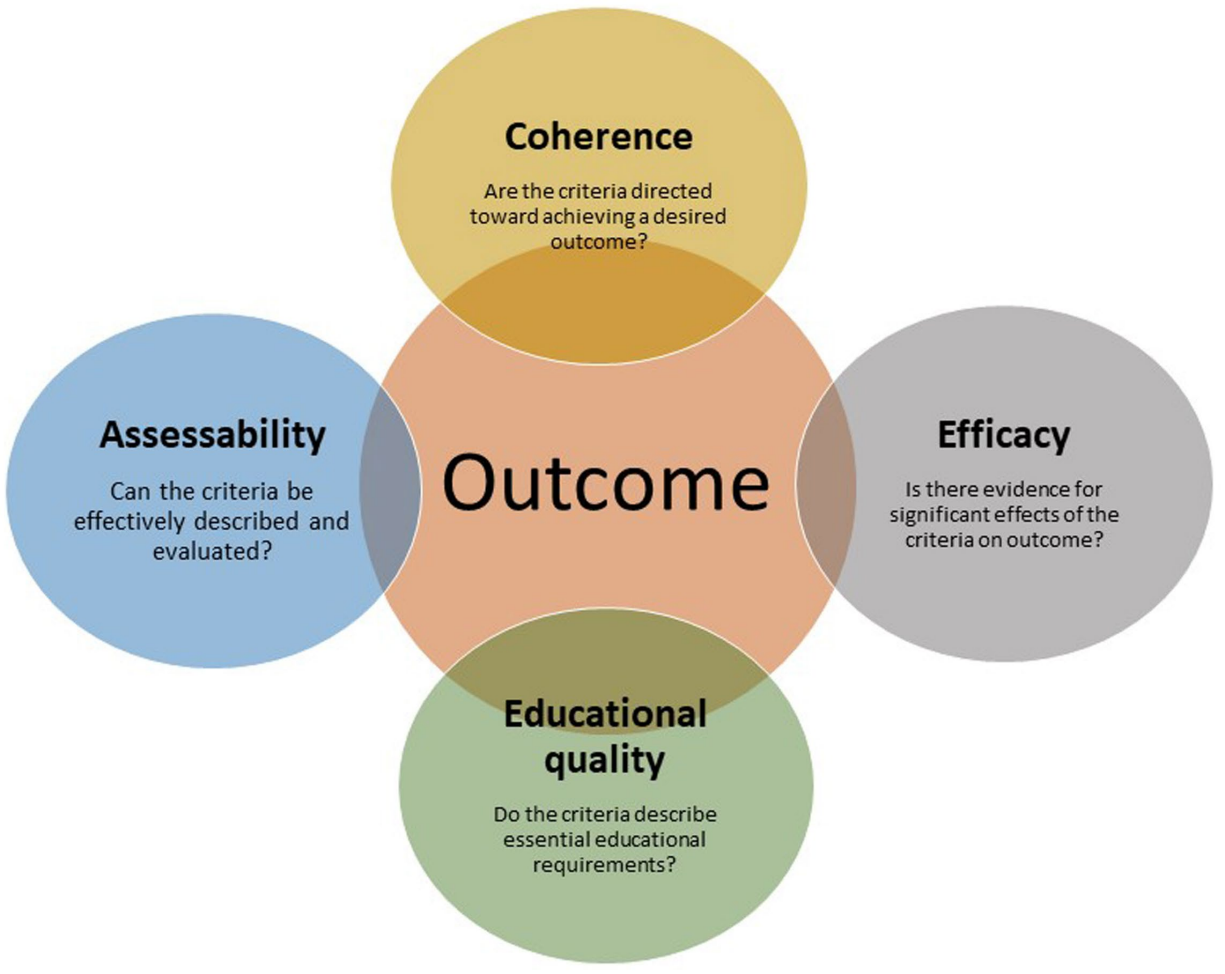
Outcome-directed justification principles for selection of accreditation criteria.

In this section, a framework for principles that guide the selection of CE accreditation criteria is presented. The justifications for criteria selections will be discussed in view of higher education research and accreditation standards established earlier by other associations or authorities. The focus is on criteria that are essential for CE quality evaluation, and the main justifications for selection of those criteria are outlined in Figure 1. Meaningful accreditation criteria are directed at essential educational requirements (Principle 1), coherence (Principle 2; defined by Main and Pendergast (17) as the connection between the professional development activity and the practical outcome of CE), and efficacy (Principle 3; described in section 5.2.). In order to make any reviewing process feasible and practical, the criteria should also be possible to describe and to assess (Principle 4).

Selected criteria should ideally fulfill all of these justification principles. Criteria that do not correspond to the justification principles may prove redundant and unnecessarily time consuming in an accreditation process, or may not be adequately supported by scientific evidence. There is, however, limited evidence on the sensitivity or specificity of accreditation criteria in recognizing deficient or ineffective CE in advance, and hence a quality control cycle with feedback and constant adjustments within a self-regulatory assessment system is vital in this context. As there are limited mechanisms for veterinary licensing bodies to assess whether CE will have the desired effect on professional development after graduation (2), re-evaluating and regulating CE programs is relied upon to ensure effective learning.

### 5.2. Efficacy and outcome of CE

Effectiveness of teaching remains difficult to measure or report. There is a lack of consensus on what constitutes effective CE, but a hierarchical structure of outcome assessment has been suggested as a useful framework for veterinary CE. This assessment, in increasing order of significance, consists of participant experience and feedback, participants’ achievement of learning outcomes, organizational support for change and application of new knowledge, achieved change and use of new knowledge, and improved clinical outcomes (2, 18, 19). Similarly, an outcome-based hierarchy for evaluation of educational programs, where levels of learning were depicted firstly as behavioral intentions, then actual incidents of changed behavior, and lastly, changes in behavior leading to benefits for the practice, clients, or animals (20, 21). These elements are part of knowledge translation, with its knowledge creation cycle and action cycle (22, 23), where an effective learning process is actually transferred into actionable outcomes and effects on clinical practice.

A direct association between CE and practical outcomes is hard to verify with validity and precision (24), especially if this evaluation needs to be done in advance for accreditation purposes. Mechanisms that drive successful outcomes (i.e. behavior change and improvement in practice) include developing skills in reflection, self-awareness, and self-assessment, as these support continued self-directed learning. Outcomes are also strongly affected by workplace culture and organizational values. Nurturing a culture of inquiry, learning, and implementation, and focusing on team competences rather than individual skills have a great impact on the consequences of CE (24), and the availability of accredited, high quality courses could increase the employer confidence in CE and maintain a positive learning culture. Training on self-directed continuous learning could also be a part of a team’s CE plan, together with other generic skills.

### 5.3. Core features of accreditation criteria

Main and Pendergast (17) made an effort to develop an evidence-based tool for designing and delivering effective CE for middle level school teachers. The tool identifies five core features of effective CPD: content focus, active learning, coherence, duration, and collective participation. These elements are also included in the accreditation criteria discussed in Part 2 of this paper. More elaborately defined high-quality elements have been described in the Accreditation with Commendation criteria menu created in 2016 by the ACCME (12, 25), where 16 selected criteria were included, divided into five categories: team-based education, public health priorities, skill enhancement, educational leadership, and achievement of desired outcomes. To be eligible for Accreditation with Commendation, providers need to present evidence for at least eight of the mentioned criteria. In a five-year follow-up on the new commendation criteria, McMahon (25) reported that educational organizations had accepted the challenge and worked toward fulfilling these criteria in addition to the minimum requirements.

In 2016, a group of international leaders in accreditation proposed a set of core principles for all medical CE accreditation systems to use as the basis for their evaluation (26). These core principles are: “*Learning activities are developed to address the needs and professional practice gaps of members of the target audience; the content is informed by evidence and bias is minimized; learning activities are designed efficiently to maximize educational impact; learning activities are planned and managed to ensure independence from external interests; there is a rigorous evaluation of educational outcomes including how education has impacted knowledge, competence, performance, and health outcomes; the accreditation standards and processes are consistently and fairly applied and continually enhanced*.”

### 5.4. Sources and selection of criteria

The categories of criteria used in medical CE are quite readily adaptable and useful also in veterinary CE, although the diversity of veterinary work calls for careful consideration of additional criteria. The VetCEE organization has established its set of accreditation criteria based on the published criteria and experiences of other accrediting bodies (10–12, 14, 25, 26) and on the pedagogical research described in this text, and made final selections based on the four justification principles described in section 5.1. The selected accreditation criteria can be divided into 3 categories (Table 1), and are discussed in more detail in section 6. Literature searches for this review were done on ERIC (ProQuest), PubMED, SpringerLink, Google Scholar, and Scopus, using search terms relevant to each section. References were also retrieved via articles found in these databases.

**Table 1:** Accreditation criteria of the Veterinary Continuing Education in Europe (VetCEE).

Category of accreditation criteria	Subject of criteria
Educational quality	Needs assessment and purpose
	Definition of target audience and level of instruction
	Definition of scope, workload, and number of credits
	Organizer and instructor qualifications
	Constructive alignment
	Assessment
	Learner engagement
	Scientific quality and evidence-based medicine
Procedural elements	Completeness of submitted information
	Adherence to submitted and accredited course plans
	Feedback mechanisms and quality assurance
General policies	Animal welfare and ethical use of animals
	Compliance with ethical and legal requirements
	Environmental sustainability
	Equality and diversity
	Commercial influence and bias
	Conflict of interest

## 6. Selected accreditation criteria

### 6.1. Needs assessment and identification of purpose in CE

A clearly identified educational need sets the stage for a positive learning outcome, as it is an essential part of self-directed learning (27). The desired outcome, in fact, defines the need and forms the whole basis for planning CE with its purpose, activities and assessments. The learning process is enabled when an educational need has been identified and is clearly linked to clinical practice, and in the ACCME model, the CPD process has been described to begin with the practitioner asking a question and end with an evaluation of the impact of an implemented, CPD-induced change (11). A conceptual change in the internal models, or schemas, is the foundation of learning. In order for a conceptual change and accommodation to occur, these internal schemas need to be identified and challenged, otherwise the learner is subject to a restricted but comfortable level of confirmation bias and limited assimilation of new knowledge (28, 29). The prerequisite for a successful education outcome is the ability of the practitioners to critically evaluate their current practices, challenge their previous knowledge, and identify a need for improvement.

An educational need may be identified either by the learner or suitable areas suggested by CE providers, but in the context of planning CE in formal course form, the responsibility tends to lie on the providers. In medical CE, providers are required to ensure that identification of educational needs are based on analyses and that these needs are also documented and used for content development (30). The methods of needs assessments in medical CPD have been reviewed by Grant (31). In order to identify useful topics, providers can use for instance user questionnaires or surveys, expert advice, scientific literature, or veterinary training program content descriptions. The medical field presents a perhaps more cohesive audience than the veterinary one, with certain competencies reserved for CE or specialist training programs. With the marked diversity in veterinary practice, there are often no specific, universal topics that CE needs to address, and therefore decisions on themes and contents in veterinary CE are, in general, left to providers. Specialist programs usually have specified learning outcomes, and these can serve as guidance for content planning.

In the end, the decisions on what CE topics to focus on is largely at the discretion of the individual practitioner. Providers, licensing bodies, mentoring systems, or accreditation organizations could, however, play a significant role in assisting practitioners in identifying areas of improvement and planning their individual CPD, as the individual veterinarian may not be accurately assessing their own skills (2). Competence of veterinary professionals can be evaluated via formal assessments by licensing bodies, self- and peer evaluation in clinical audits, or recognized via feedback from clients, but overburdening of practitioners should also be avoided (2). Accreditation bodies could support practitioners and providers by specifically rewarding providers that assist individuals in their self-assessments (11) or provide comprehensive training programs, or by building CE plan templates for different career stages or fields of veterinary medicine.

A defined curricula for veterinary development has been proposed (32) but not commonly applied, perhaps due to the diverse educational needs of veterinary practitioners. It appears that CE is more likely to be effective when individuals actively create their own CE plans according to their own needs. There is, however, a mismatch between topics considered high priority (such as treatment of specific medical conditions), and the topics that are ultimately chosen and attended (such as CE on regulatory compliance) (2, 33, 34). Gates et al. (2) suggested that templates providing guidance on self-assessment could be created to support practitioners in their CE decisions, and the authors also emphasized the value of external feedback from peers, mentors, or licensed professionals.

While a survey by Ebell et al. (35) showed that the most common questions veterinarians ask in practice relate to use of specific drugs or treatments, other key areas of knowledge have emerged. Competency to practice veterinary medicine has been defined to include not only clinical knowledge and technical skills, but also the competence domains of critical reasoning, professional identity and personal well-being. It has been suggested that personal well-being assessments and training should be incorporated into required and regulated CE, as burnout is a common problem among veterinary professionals (2).

In conclusion, identification of a need and purpose of CE fulfills the principles mentioned in section 5.1, represents one of the main criteria in medical CE accreditation (30), and was therefore selected as one of the accreditation criteria for veterinary CE. Needs assessments are fundamental for learning, and for CE selection by individual veterinarians and development of CE programs by providers (Principle 1). They are also coherent, i.e. there is a connection between the professional development activity and the CE outcome in clinical practice (17). In addition, needs assessment is useful and practical (Principle 2 and 4), as the perceived needs are often aimed directly at improving clinical practice or other vital skills, and will help focus limited time resources effectively. The efficacy (Principle 3) of identifying group and individual learning needs is still in need of further research, and the effects of needs assessment must be considered in a wider context of the learning process (31). The development and outcome of different needs assessment methods also need further research, as reporting deficiencies have made challenging to assess the efficacy of different methods (36).

### 6.2. Definition of the target audience and suitable level of instruction

Definition of the target audience is closely related to needs assessment, and specifies for whom the CE event is planned. The requirements of CE directed at board-certified experienced specialists can be markedly different from that designed for recent veterinary graduates, and this knowledge diversity needs to be taken into account. Veterinarians at different career stages may also have many common areas of interest or needed improvement, but considering these differences during CE planning is needed in order to adapt the level of instruction accordingly. Learning activities may also benefit from having several layers of complexity to serve the needs of practitioners at different career stages (37), and it encourages productive discussion between these groups. Early-career veterinarians need CPD for support in establishing basic clinical skills and confidence, but similar levels of instruction may be appropriate for mid- or late-career stages after career breaks or CPD gaps (2), and a competency-based approach may be more descriptive than time from graduation.

While the Day One Competencies at graduation have been discussed extensively in veterinary schools, recent graduates most likely require additional experience and practice to truly attain those competencies (2). A competency-based model of CPD takes a dynamic view on the process and contexts of professional development over time, and emphasizes competencies that are related to self-directed learning (37). Individual veterinarians are largely themselves responsible for building their own personal CPD, which can develop into pathways that eventually lead to a specialist degree. The European Qualifications Framework (EQF) serves as a guide for translation of qualifications from different countries and institutions (38), and is a useful aid for the description of target groups and level of instruction, enabling the selection of appropriate CE levels. Matching specific competencies with EQF levels is an ongoing debate with variation between countries, but certain field-specific guidelines can be attained from specialist training program learning outcome descriptions or discipline-specific competency templates issued by licensing authorities. Competence is also not static or permanent, but instead graduates also need to sustain and renew their graduation level skills (37).

As a fundamental building block of CE planning, definition of the target audience and level of instruction fulfill all of the mentioned selection principles, and should be included as a main criteria for CE approval. The VetCEE criteria state that courses should be directed at a suitable target audience, and that the level of instruction should correspond to the purpose of the course, and the level and prerequisites of the target audience (16).

### 6.3. Provider and instructor qualifications

With the increased demand for CE comes increased variation in provider and instructor qualifications. The role of an accreditation organization is to provide licensing bodies, employers, and individual veterinarians with a reliable assessment of these qualifications. Assessment of providers rather than activities is performed in medical CE accreditation, but the accreditation systems also relies on data collection, program evaluations, and reporting on specific activities by providers (10, 12). The ACCME accreditation also has requirements regarding the provider’s planning process, organizational framework, and business and management practices (10), but not specifically regarding instructor qualifications, perhaps because of marked diversity in instructor and provider competencies and career paths.

The assessment of adequate competence in education and the specific field of instruction is not well described in accreditation criteria of medical CE. In veterinary CE, the German ATF criteria include the following criteria regarding provider and instructor qualifications: “*Speakers must have practical and academic experience that qualifies them to teach the topic covered in the segment, course, or module. The host is reliable and trustworthy and will conduct the course faultlessly*.” (39). More detailed assessment of what is deemed adequate is left to the assessor. The AAVSB states in their RACE standards for veterinary CE that instructors must be knowledgeable in the subject at a level higher than the intended audience, and have specialized training, experience, and knowledge in the subject matter, and these qualifications are described in more detail (14). The emphasis of instructor qualification requirements tend to lie on subject matter competencies, while educational scholarship is hardly mentioned. Medical and veterinary CE educators often have limited time resources for further training in educational matters. Fragmentation of time to work on educational projects, prioritization and competing responsibilities, and lack of motivation and prestige interfere with pedagogical CE of medical and veterinary educators (40).

The VetCEE criteria currently state that the instructors’ competencies should be appropriate and sufficient in order to meet the educational needs and to achieve the intended learning outcomes. Detailed qualification requirements could be of use, but are difficult to describe for all possible scenarios. There is also a requirement regarding the number of instructors, as an adequate number of instructors are needed to meet the educational needs and to achieve the learning outcomes, especially in practical course work (16).

### 6.4. Constructive alignment: learning outcomes, activities, and assessment

#### 6.4.1. Definitions and concerns

Constructive alignment (CA), with its principles of aligning intended learning outcomes, constructive student-centred learning activities, and assessments (41), is one of the fundamental pillars of higher education and curriculum planning, and lies under the umbrella term of outcomes-based education, (42). The use and importance of CA in higher education is widespread, but it has been argued that an emphasis on alignment and learning outcomes in validation and audit purposes creates an illusion of quality control with little practical meaning (42). Biggs (41) highlighted the importance of student-centered learning activities (what the student does), whereas demonstration of alignment purely for QA validation defeats its purposes. With criticism directed at constructivist theory in science education concerning limited guidance of students (43) or maintenance of faulty constructs (44), terms such as “curriculum alignment” and “curriculum congruence” may be more accurate or preferable (44, 45).

Accreditation systems and other quality assessments in medical CE commonly refer to CA principles for course evaluation, but are more poorly defined in veterinary CE. In the ACCME medical accreditation system, planning processes of accredited providers are required to include matching educational content to desired results, communicating intended learning outcomes to learners, and measuring the satisfaction, knowledge, or skill of the learner at the conclusion of the educational activity (12). On a European level, alignment principles are outlined in the EQF, the ECTS Users’ Guide (46), and the Guidelines for Quality Assurance in the European Higher Education Area (47), but these principles need to be implemented in practice and not only symbolically documented. The role of the accreditation body is therefore to assess the actual fulfillment of these principles, and prognosticating these outcomes in advance is challenging. Evaluation of CE outcomes, with true integration of newly attained skills into clinical practice as measures of success, could serve as building blocks toward accreditation with commendation or a “Trusted provider” status. The focus of CA needs to lie on teaching and learning, and not on documentation for quality assurance (42, 48).

#### 6.4.2. Start with the end in mind – competency- or outcome-based approaches

Medical and veterinary CE have both evolved toward an outcome-based approach, but still today CE often provides little more than documentation of attendance, serving a limited purpose for improvement in practical terms, such as in patient outcomes (49). Traditional veterinary CE is often teacher-centered with minimal interaction between learners and providers, with emphasis on attendance and credit or certificate requirements (50). Learning in CE should instead be designed to achieve quantifiable improvements in practice (37). The planning process in a competency- or outcome-based learning event starts with the end in mind and works backward, from needs assessments to intended learning outcomes, activities and assessment (49). In a clinical CE context, planning should incorporate also considerations of career and learning stages of the participants, a clear focus on practical, clinical problems with meaningful implementation opportunities, and provision of practice and feedback in authentic settings (49).

Measurement of outcomes should go beyond satisfaction of learners or self-reporting of learning, and instead measure higher levels of outcomes (37). Moore et al. (49) made an effort to build a conceptual model to guide medical CE planning in order to achieve better outcomes. This could be done through integrating assessment in the instructional design continuously throughout the learning activities, and focus planning and assessment on achieving desired outcomes. The authors suggested considering outcome levels above declarative and procedural knowledge, including outcomes such as performance of new skills, and improvement in patient and community health.

#### 6.4.3. Teaching and learning activities: learner engagement, social, and active learning

Active learning models should be preferred and supported in accredited CE. Active learning involves opportunities to be actively engaged in meaningful discussions, planning, and practice during CE (17). The notion that education is an ‘active and constructive process’ (51) can guide choices of educational methods and activities, all the while considering the diversity in skills and backgrounds of the participants (50). Learning by doing, ideally under the guidance of an instructor or mentor, together with formative assessment, is considered one of the most effective forms of veterinary CE (50). Passive approaches to learning have not been effective in changing physician behavior in medical CE (52), while interactive and engaging models with reflection on current practices, knowledge gaps, and practice of skills tends to lead to better and broader outcomes (53). Actually demonstrating newly learned skills, and not only having the “know-how,” but also being able to “show-how,” displays a higher level of learning through cycles of practice and feedback, or the instructional framework of predisposing-enabling-reinforcing that should not be omitted (49).

The format of CE does not appear to affect outcome (2, 54, 55), and course format has not been considered a threshold requirement in CE accreditation, although conferences and workshops are not very likely to have significant impact on clinical performance (56). Traditional lecture-based and information transmission format CE appears to only rarely result in meaningful changes in behavior despite transfer of some factual knowledge (reviewed by (21)). Multifaceted activities that combine several different interventions have been shown to be effective (57). Development of new models of CE are needed, and are certainly enhanced by the development of digital teaching technologies. Monitoring achieved credits and professional development can also be achieved via web-based platforms and applications.

Development of skills in reflection has become a central theme in modern CE, as it is considered a vital component of personal and professional development (50). Reflective practices can serve a function in self-assessment, identification of knowledge gaps, and recognition of professional development. There are different models for the use of reflective thinking and learning, but they are mostly based on the principle that ‘individuals need to be able to describe what they are currently doing, how those practices impact both themselves and others, and what (if anything) they will change about their current approach to improve outcomes the next time’ (2). The skills of reflection take considerable practice to develop, and mentoring can be of great help.

Teaching strategies facilitating true learner engagement are essential. Successful CE is designed based on practical needs to facilitate individualized change (11), based on engaging the learners through providing training that they find immediately useful in their daily work, and hereby enhancing their interior motivation. It has been widely recognized that mere attendance of CE events, such as conferences or rounds, are no guarantee of learning or improvements in professional practice (21, 56, 58).

Social aspects of learning have been emphasized increasingly in the past decades. Interactions with peers to support professional development is a part of general qualitative themes on effective CE in the literature (2), but comparisons between group and individual learning within the context of medical or veterinary CE is non-existent or conflicting. Campbell et al. (37) summarized systematic reviews on the effectiveness of group learning, and concluded that group learning had a moderately positive impact on knowledge, but only a small to negligible impact on clinical behaviors and patient outcomes. In contrast, other authors, such as Main and Pendergast (17) and Parboosingh et al. (59) have concluded that interaction between participants provided essential improvements in practice. Similarly, Regnier et al. (11) stated that small group interaction provides opportunities for analysis and synthesis on knowledge into new strategies to be implemented in practice. Practitioners may definitely benefit from the peer support in social learning, as it can encourage mutual change and decrease feelings of loneliness and hesitancy in incorporating newly acquired skills into daily practice.

#### 6.4.4. Assessment

Assessment strongly affects the students’ learning processes (60, 61), and this is also a major factor in CE assessments. Assessments are in general required for accreditation, but should be performed with an educational aim in mind, and not to simply fulfill accreditation criteria. Assessment should serve a clear educational purpose, instead of only being a way of meeting requirements and proving attendance. Knowledge retention and outcome of CE are often improved when participants are formally assessed on their learning, as self-assessments are often biased and misguided (2).

Assessments also give the provider information on the effectiveness of the event and identification on additional educational needs. Ideally, assessments also measure improvements of higher outcome levels, such as work performance and patient outcomes in the long term (30). While one function of assessments has traditionally been to ascertain the achievement of specific learning goals, assessments also serve the important purpose of guiding learning processes and directing and motivating the students through feedback (62).

The addition of self and peer review aspects to assessments can support learners in their ongoing self-evaluation in daily work, and such assessment for learning practices and critical analysis are a vital part of lifelong learning and sustainable assessment (63, 64). The methods of assessment should support work life needs, and involve the student as an active participant (63, 65, 66). Assessments should also be reliable, fair and valid (67), and the objectives, content, and knowledge levels of the assessed learning outcomes should be communicated to the students well in advance (60). In practice, the method of assessment can take many forms, as long as the mentioned basic principles are considered.

### 6.5. Evidence-based medicine, scientific quality, and evaluation of content

Evidence-based medicine (EBM) is an integration of the best evidence into clinical expertise, with further consideration of a patient’s values or preferences (68). To maintain its image of accountability and reliability, high quality veterinary CE needs to be scientifically sound, and CE provides an avenue for translation of research into practice. Evaluation of the content and level of evidence requires extensive subject expertise, and a peer review system similar to the review of research papers is a practical approach in this respect. At best, CE also teaches critical assessment of evidence in association with the subject at hand.

An emphasis on clinical skills and evidence-based medicine are core features of effective CE according to the study by May and Kinnison in 2015. The Cologne Consensus Conference Standards and Guidelines in Accredited CPD in 2019 (69) discussed criteria for evaluation of medical CE content, and concluded that CE content should be “*based on evidence that is accepted within the profession as scientifically valid, adequate to justify recommendations related to the care of patients, balanced in the review of all relevant options, provided by individuals whose conflicts of interest are appropriately managed and resolved, and comprehensive in scope, addressing the range of competencies relevant to the provision of safe, high quality healthcare.”* In medical CE accreditation, CE content is expected to represent the latest advances in scientific evidence and technological advances relevant to the practice of medicine. Furthermore, content should be relevant and balanced, informing learners about potential benefits and risks, especially if the content is based only on expert opinion. Freedom from commercial bias is also a basic requirement (30).

### 6.6. Scope, workload, and credits

The ECTS User’s guide (46) provides directions for allocation of credits according to workload, and these are well established in Europe. With regard to accreditation of CE, the alignment of between workload and credits is evaluated. The amount of work should be feasible within the particular credit and time framework. Course feedback is essential, as adjustments may be necessary if the same course is repeated. Duration and scope of courses can vary markedly, and this variation is an advantage as it provides a broad supply of CE to meet different needs. According to a questionnaire by Huntley et al. (70), the preferred method of CE for veterinarians were day or evening seminars, conferences, and online courses (49.7, 23.4, and 8.7% of respondents).

The duration of courses varies, but should be of “*sufficient duration to enable engagement, leading to possible intellectual and pedagogical change”* (17) for the particular subject and at the desired level. O’Brien (71) suggested that the most effective professional learning programs consisted of multiple days, over a period of time, but the demands of modern work life and the technological advances has brought the CE field a multitude of options.

### 6.7. Ethical conduct and policies

Ethical conduct and adherence to all relevant legislation are an indisputable requirement of accreditable CE. An in-depth discussion of ethical policies is beyond the scope of this review, but independence and transparency, with disclosure of competing interests, form the basis for avoidance of commercial bias (30). Clear guidelines are needed on what is acceptable or allowed regarding the role of the industry. The Cologne Consensus Conference has listed several policies related to commercial bias, and these serve as an excellent guide for accreditors (69). Animal welfare should be a priority for all involved in teaching of veterinary medicine, and relevant regulations should be followed, with descriptions of animal use included in applications. Diversity and inclusivity in teaching and environmental sustainability are factors to take into account also in veterinary CE.

## 7. Accreditation protocol and quality assurance policy

The VetCEE accreditation standards and protocol have been published in 2022 (16). As knowledge and experience build, the accreditation criteria and protocols need reviewing and should be modified accordingly. Accreditation bodies should be subjected to regular internal and external quality assurance evaluations, coupled with the relevant implementation plans, monitoring and follow-up, as described by ENQA (47) and EQAR (72). In medical CE, it has been agreed that all actions of accreditation bodies should be open and transparent, and all rules and regulations should be clear and unambiguous, in order to avoid arbitrary decisions. Regular engagement with the profession and providers improves accreditation interest and compliance (69).

Feedback loops are an essential part of the development of not only individual CE activities, but also the accreditation processes. Feedback from CE participants of accredited activities should be collected and analyzed for key success factors, such as the critical levels of feedback information described by Guskey (73): the participants’ satisfaction and learning, the support for change by the participants’ organizations, the participants’ use of new knowledge and skills at the level of implementation, and the participants’ learning outcomes in the long term (the accomplished modifications of daily practice and outcomes relating directly to patient care). The last three levels would require collection of long term longitudinal data. While reflective practices are nowadays commonly taught to CE participants, CE providers and accreditation bodies also need to be reflective practitioners and constantly monitor, improve, and update their work.

The Cologne Consensus Conference Standards and Guidelines in Accredited CPD in 2019 described the roles and responsibilities of accrediting bodies in medical CE (69). They conferred that accreditors must have developed and implemented policies and procedures regarding the following elements: eligibility for accreditation; description and implementation of the reviewing process, standards, and appeal processes; communication of requirements and processes to applicants; requirements of improvements in case of less than full compliance by providers; and retainment of records according to applicable national regulations. Accreditation should occur independently from any third party influence and not involve any commercial benefit (69). These and other elements are included in the VetCEE process description in the VetCEE Standards and Protocols.

## 8. Discussion

Accreditation criteria for veterinary CE were selected based on four selection principles: educational requirements, coherence, efficacy, and assessability. The main accreditation criteria for veterinary CE include a needs assessment and identification of a clear purpose for the activity. Definition of the target audience, suitable level of instruction, and scope all together form a starting point for CE planning and should be taken into account during evaluation. The provider and instructor qualifications provide a basis for activities with robust scientific quality. The basic tenets of constructive alignment are still valid and useful, as long as they are truly implemented and not only symbolical. The significance of assessment in guiding the learning process accentuates the importance of planning suitable assessment tasks. Ethical conduct and adherence to all relevant regulations and ethical policies are vital for maintaining reliability and accountability in veterinary CE. The accreditation workflow needs to be easy to complete, in order to encourage providers to participate.

Accreditation criteria and protocols need to be regularly re-evaluated and modified in close collaboration with the relevant stakeholders. Feedback loops and their associated implementation plans bring about improvements of evaluation systems. When CE activities, not providers, are accredited, there is room for flexibility, growth, and development, but long term follow-up of learning outcomes may prove challenging. The accreditation criteria need to be clear, and openly communicated to all parties, to ensure fair and unambiguous decisions.

The desired outcome of CE, including behavior change and improvement of practice and ultimately human and animal health, remains challenging to predict based on course descriptions by the providers. A Trusted Provider status could be an option for rewarding commendable education practices with documented positive outcomes, in a similar manner as the ACCME Accreditation with Commendation (12). Accreditation bodies or providers are not alone in making CE effective, however, and the workplace has its own role as it provides opportunities for CE and can nurture a culture of inquiry, learning, and implementation of new skills. A focus on team competencies and active sharing of knowledge can support veterinary CE, and especially the long term, practical effects of learning (24).

The role of a common European accreditation service, such as VetCEE, has yet to be established, and promoting this pan-European accreditation service is currently under way. The stakeholders will eventually direct the functions of a common accreditation body according to the needs of the veterinarians and national competent authorities regulating veterinary specialization qualifications. Accrediting bodies in veterinary medicine should create stronger collaboration globally and in conjunction with accreditation systems in human medicine, in order to develop their services further. Accreditation bodies also need to accept their responsibilities within the field of education, leading the way toward lifelong learning, flexible career pathways, and scientific integrity. According to McMahon (74), accreditation bodies can facilitate innovation through actively re-evaluating their requirements for maximum flexibility, while also maintaining quality, integrity, and educational independence. Accreditation bodies can also stand up for science and counter misinformation. Peer support and lifelong learning can also help veterinarians stay in the profession, preventing burnout and promoting rewarding professional development.

## Author contributions

MK conceptualized and wrote the manuscript.

## Conflict of interest

MK is affiliated with VetCEE and the University of Helsinki. She has no other financial interests or benefits to disclose. Views and opinions expressed in this text are those of the author and do not necessarily reflect the views or positions of any entities they represent.

## Publisher’s note

All claims expressed in this article are solely those of the authors and do not necessarily represent those of their affiliated organizations, or those of the publisher, the editors and the reviewers. Any product that may be evaluated in this article, or claim that may be made by its manufacturer, is not guaranteed or endorsed by the publisher.
